# Enhanced Intrusion Detection for ICS Using MS1DCNN and Transformer to Tackle Data Imbalance

**DOI:** 10.3390/s24247883

**Published:** 2024-12-10

**Authors:** Yuanlin Zhang, Lei Zhang, Xiaoyuan Zheng

**Affiliations:** School of Artificial Intelligence and Data Science, Hebei University of Technology, Tianjin 300132, China; 202232804026@stu.hebut.edu.cn (Y.Z.); 2021019@hebut.edu.cn (X.Z.)

**Keywords:** industrial control security, network security, intrusion detection, data imbalance

## Abstract

With the escalating threat posed by network intrusions, the development of efficient intrusion detection systems (IDSs) has become imperative. This study focuses on improving detection performance in programmable logic controller (PLC) network security while addressing challenges related to data imbalance and long-tail distributions. A dataset containing five types of attacks targeting programmable logic controllers (PLCs) in industrial control systems (ICS) was first constructed. To address class imbalance and challenges posed by complex network traffic, Synthetic Minority Oversampling Technique (SMOTE) and Borderline-SMOTE were applied to oversample minority classes, thereby enhancing their diversity. This paper proposes a dual-channel feature extraction model that integrates a multi-scale one-dimensional convolutional neural network (MS1DCNN) and a Weight-Dropped Transformer (WDTransformer) for IDS. The MS1DCNN is designed to extract fine-grained temporal features from packet-level data, whereas the WDTransformer leverages self-attention mechanisms to capture long-range dependencies and incorporates regularization techniques to mitigate overfitting. To further enhance performance on long-tail distributions, a custom combined loss function was developed by integrating cross-entropy loss and focal loss to reduce misclassification in minority classes. Experimental validation on the constructed dataset demonstrated that the proposed model achieved an accuracy of 95.11% and an F1 score of 95.12%, significantly outperforming traditional machine learning and deep learning models.

## 1. Introduction

Industrial control systems (ICSs), which integrate hardware and software to manage industrial equipment and processes, are extensively employed in sectors such as energy, manufacturing, and chemical industries [1]. A key component of ICSs is the Programmable Logic Controller (PLC), which serves as a critical automation device for monitoring and controlling physical processes [2]. These systems collect real-time data and enable remote control. By facilitating intelligent decision-making, they enhance both operational stability and efficiency [3]. However, the growing connectivity of ICSs has exposed these systems to a variety of cybersecurity threats, including cyberattacks, malware, and insider threats, which pose significant risks to both operational stability and safety. For instance, the 2015 cyberattack on the Ukrainian power grid caused a widespread blackout, underscoring the vulnerability of ICSs to targeted attacks [4]. In 2019, a gas facility in the United States fell victim to a phishing attack [5]. In 2020, a cyberattack caused severe damage and explosions at a Ukrainian chemical plant, emphasizing the urgent need for robust ICS security [6].

A critical challenge in intrusion detection for ICSs is addressing data imbalance [7] and long-tail distributions [8] in network traffic data. Data imbalance arises when the number of attack samples is significantly smaller than that of normal samples, making it challenging for models to effectively identify rare attack types. Furthermore, some attack categories exhibit a long-tail distribution, where the majority of data are concentrated in a few dominant classes, leaving others underrepresented. This imbalance and the sparsity of minority classes intensify the challenge in achieving accurate detection, especially in industrial scenarios characterized by diverse attack patterns.

Machine learning and deep learning techniques provide substantial advantages for intrusion detection, particularly in identifying unknown attacks and addressing complex threats [9]. Deep learning models are capable of learning the patterns of normal and abnormal behaviors from data, thereby enabling automated threat detection. These methods have demonstrated outstanding performance in various domains, including computer vision, speech recognition, and industrial safety analysis. Deep learning-based intrusion detection systems (IDSs) enhance detection accuracy and adaptability, contributing to the protection of critical devices, such as PLCs, against evolving cyber threats [10].

To address these challenges, this paper presents a novel approach aimed at enhancing PLC network security through innovative methodologies and models. The main contributions of this study are outlined as follows:

1. Proposal of the Weight-Dropped Transformer (WDTransformer): An enhanced Transformer encoder architecture that incorporates dynamic sparsity using DropConnect, a generalized regularization method that probabilistically removes weight connections during training;
2. Development of a hybrid dual-channel feature extraction model: This model combines multi-scale one-dimensional convolutional neural networks (MS1DCNN) and the WDTransformer. The MS1DCNN module extracts fine-grained temporal features across multiple scales, while the WDTransformer module emphasizes capturing global dependencies with improved regularization;
3. Creation of a custom dataset for PLC network security in ICSs: This dataset encompasses diverse attack scenarios such as SYN-Flood, DDoS, ARP, and Nmap attacks to ensure realistic and comprehensive evaluation.

The remainder of this paper is organized as follows. Section 2 reviews related work on intrusion detection for ICSs. Section 3 elaborates on the proposed methodology, including data preprocessing, oversampling techniques, and the design of the dual-channel feature extraction model. Section 4 presents the experimental results and their analysis. Finally, Section 5 concludes the study and suggests directions for future research.

## 2. Related Works

In recent years, network intrusion detection has emerged as a prominent research domain. A variety of deep learning approaches have been proposed to tackle increasingly complex threats. Current studies encompass various model architectures, including convolutional neural networks (CNNs) [11], Recurrent Neural Networks (RNNs) [12], and attention mechanisms [13]. These studies employ diverse techniques for feature extraction and temporal modeling, with an emphasis on feature selection and data balancing to enhance model performance. This section provides a review of key studies in intrusion detection, focusing on their methodologies, contributions, and limitations.

Recent research has explored hybrid frameworks to capture both temporal and spatial features. For example, Asmaa Halbouni et al. [14] combined CNN and Long Short-Term Memory (LSTM) networks for intrusion detection using three public datasets: CIC-IDS2017, UNSW-NB15, and WSN-DS. However, their approach relied on general network traffic data without testing in specific industrial environments, limiting its applicability. Similarly, Mohammad Mehedi Hassan et al. [15] proposed a hybrid CNN and Weight-Dropped LSTM (WDLSTM) model to extract spatial features and maintain long-range dependencies while mitigating overfitting. Although the model demonstrated improved performance on public datasets, it did not address data imbalance, a critical issue in real-world scenarios. Additionally, Kai Jin et al. [16] developed a multi-scale CNN (MS1DCNN) model with efficient channel attention and BiLSTM layers for feature extraction. While the model’s hyperparameter optimization enhanced classification performance, the use of SMOTE for data balancing introduced noise, reducing its generalization capabilities for minority classes.

Transformer-based methods have gained traction for their ability to capture long-range dependencies in network traffic. For instance, Ruizhe Yao et al. [17] applied a hybrid CNN–Transformer model to extract spatial and temporal features from network traffic data, achieving competitive performance on KDDCup99, NSL-KDD, and CIC-IDS2017 datasets. However, their use of ADASYN for data balancing introduced noise by deviating from the true data distribution. Zihan Wu et al. [18] introduced a Transformer model with stacked encoder–decoder networks to balance feature extraction and dimensionality reduction. While this model reduced noise and retained useful information, its reliance on dimensionality reduction limited its ability to model temporal dependencies directly. Similarly, Zhenyue Long et al. [19] proposed a Transformer-based intrusion detection model for cloud environments, achieving over 93% accuracy. Despite its high performance, the model struggled with extracting multi-scale features and was prone to overfitting on imbalanced datasets.

To enhance feature extraction further, several works have investigated advanced CNN architectures. Amir El-Ghamry et al. [20] combined VGG16, Inception, and Xception architectures to enhance the diversity and depth of feature extraction. While effective for spatial features, their model lacked the ability to capture temporal relationships in network data. Yanmiao Li et al. [21] proposed a multi-CNN fusion approach, converting network traffic into grayscale images for feature extraction. However, this method also struggled to model temporal dependencies, which are crucial for accurate intrusion detection.

Recent advancements in the field have further expanded the methodologies for intrusion detection. Booij et al. [22] conducted a comprehensive analysis of the heterogeneity in IoT network intrusion datasets using the ToN_IoT dataset. Their study emphasized the importance of standardizing feature descriptions and attack types to improve cross-training performance across diverse datasets. Additionally, they highlighted the need for a joint effort between the research community and industry to establish standardized benchmarks for intrusion detection. Yu et al. [23] introduced a Deep Q-Network-based open-set intrusion detection framework tailored for the Industrial Internet of Things (IIoT). Their solution leverages reinforcement learning to address the challenges of unknown attack detection in IIoT environments, achieving superior performance on the TON-IoT dataset. Thakkar and Lohiya [24] proposed a novel feature selection technique for deep neural network-based intrusion detection systems, employing a fusion of statistical importance measures such as standard deviation and median difference. Their approach was validated on multiple datasets, including UNSW-NB15 and CIC-IDS2017, demonstrating significant improvements in feature discernibility and overall performance.

Previous studies have shown limitations that highlight the need for a comprehensive approach. This includes integrating robust feature extraction, effective data augmentation, and advanced deep learning techniques. To address these challenges, this study proposes a dual-channel feature extraction model. The model combines MS1DCNN for diverse feature extraction and WDTransformer for capturing long-range dependencies. Hybrid oversampling techniques are also introduced to handle data imbalance. Unlike earlier methods, this approach achieves stable performance on both self-constructed and publicly available datasets, effectively addressing the limitations of existing techniques.

## 3. Methodology

This study develops a model that integrates MS1DCNN and WDTransformer. To address class imbalance, the SMOTE and Borderline-SMOTE algorithms were employed for dataset resampling. Additionally, a custom combined loss function was introduced, integrating cross-entropy loss and focal loss to enhance model performance on long-tail distributions. The theoretical foundation of these methods is described as follows.

### 3.1. SMOTE and Borderline-SMOTE

The SMOTE algorithm [25] addresses class imbalance by generating new samples through interpolation. Unlike random oversampling, which duplicates existing samples, SMOTE generates synthetic samples along the line segment, connecting a selected sample and its nearest neighbors. This method reduces sample overlap, thereby improving class distribution. The steps of SMOTE are as follows: determine the sampling rate N and process all minority class samples. Identify the K nearest neighbors using the Euclidean distance, then randomly select a point along the line segment connecting the selected sample and one of its neighbors to generate a synthetic sample. This method expands the dataset; the relevant formula is provided as follows:

(1) $$\left(x_{new},y_{new})=(x,y)+rand(0-1)*((x_{n}-x),(y_{n}-y)\right)$$

where (x,y) represents a minority class sample, $\left(x_{n},y_{n}\right)$ represents the coordinates of its n nearest neighbors, and $\left(x_{new},y_{new}\right)$ is the new synthetic sample generated along the line segment between the minority class sample and one of its nearest neighbors.

SMOTE treats all samples equally; however, boundary samples are more susceptible to misclassification. Therefore, focusing on boundary samples can significantly enhance classification accuracy. To address the limitation of SMOTE in distinguishing distribution characteristics, Han proposed the Borderline-SMOTE algorithm [26]. This adaptive sampling method improves upon SMOTE by concentrating on generating synthetic samples near class boundaries. Specifically, it identifies boundary samples and generates synthetic samples between these samples and their neighbors, thereby refining the decision boundary. The algorithm workflow is outlined as follows:

1. For each sample in the minority class set, find its m nearest neighbors and count the number of minority class samples among them;
2. If there are zero minority class samples, label the sample as noise, indicating that it is surrounded entirely by majority class samples. If the majority of neighbors are minority samples, label it as a safe sample, as it resides within a minority cluster. If fewer than half of the neighbors are minority samples, label it as a borderline sample, suggesting that it lies in a potential boundary region that requires special attention;
3. Apply the SMOTE algorithm to the samples marked as borderline, generating synthetic samples to balance the dataset.

The integration of SMOTE and Borderline-SMOTE leverages the strengths of both methods to comprehensively enhance model performance. First, SMOTE increases the number of minority samples across the feature space, improving data diversity and coverage. Then, Borderline-SMOTE generates samples in boundary regions, refining the precision of the decision boundary. This combination effectively reduces noise caused by class imbalance while improving the representation of minority samples and enhancing decision boundary accuracy.

### 3.2. MS1DCNN

**Figure 1:**
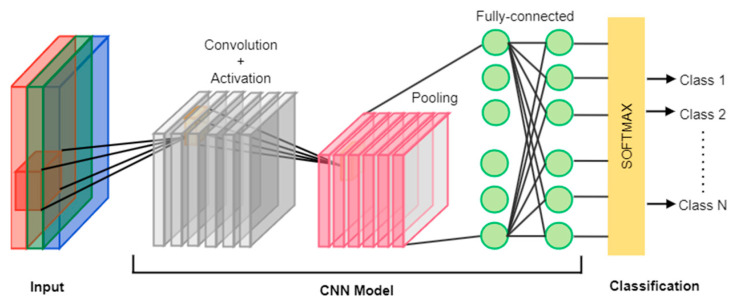
CNN structure.

CNNs primarily consist of convolutional layers, pooling layers, and fully connected layers [27]. Convolutional layers perform feature extraction, whereas activation functions introduce non-linearity. Pooling layers extract essential features and reduce output dimensions, thereby decreasing computational complexity. The overall structure of a CNN is illustrated in Figure 1. Typically, a k × k kernel is employed for two-dimensional feature extraction in CNNs.

The one-dimensional convolutional neural network (1DCNN) is particularly well-suited for processing temporal data [28]. Similar to CNNs, 1DCNNs utilize a k × 1 kernel, thereby eliminating the height dimension. Given the temporal nature of network traffic, 1DCNNs are more suitable for intrusion detection tasks due to their ability to effectively extract temporal features.

The calculation formula for 1DCNN is as follows:

(2) $$Z^{\left(l+1\right)}(t)=\sum_{i=0}^{k-1}X(t+i)W^{\left(l\right)}(i)+b^{\left(l\right)}$$

where $Z^{\left(l+1\right)}(t)$ represents the output of the convolution at time step t in layer l, X(t+i) represents the input feature value at time step t+i, $W^{\left(l\right)}(i)$ is the weight, k is the size of the convolutional kernel, and $b^{\left(l\right)}$ is the bias term.

MS1DCNN, an innovative 1DCNN neural network model [29], employs multiple 1DCNN branches with different convolutional kernel sizes to extract features across multiple time scales. Each convolution branch in MS1DCNN performs convolution operations on input data, extracting features that correspond to specific kernel sizes. The outputs from all branches are subsequently merged to form a richer representation.

The convolution process for the two parallel branches in MS1DCNN is formulated as

(3) $${Z_{1}}^{\left(l\right)}(t)=\sum_{i=0}^{k_{1}-1}X(t+i){W_{1}}^{\left(l\right)}(i)+{b_{1}}^{\left(l\right)}$$

(4) $${Z_{2}}^{\left(l\right)}(t)=\sum_{i=0}^{k_{2}-1}X(t+i){W_{2}}^{\left(l\right)}(i)+{b_{2}}^{\left(l\right)}$$

where ${Z_{1}}^{\left(l\right)}(t)$ and ${Z_{2}}^{\left(l\right)}(t)$ represent the outputs of the convolution branches with kernel sizes $k_{1}$ and $k_{2}$ at time step t in the t layer, X(t+i) is the input feature at time step t+i, ${W_{1}}^{\left(l\right)}(i)$ and ${W_{2}}^{\left(l\right)}(i)$ are the respective weight matrices, and $b^{\left(l\right)}$ is the corresponding bias term.

These branches extract features at different scales through their respective convolution operations. The resulting multi-scale features are combined using a concatenation operation. The merged features are subsequently fed into layers such as pooling or fully connected layers for final classification. This multi-scale design ensures a more comprehensive representation of the input data.

### 3.3. Weight-Dropped Transformer

**Figure 2:**
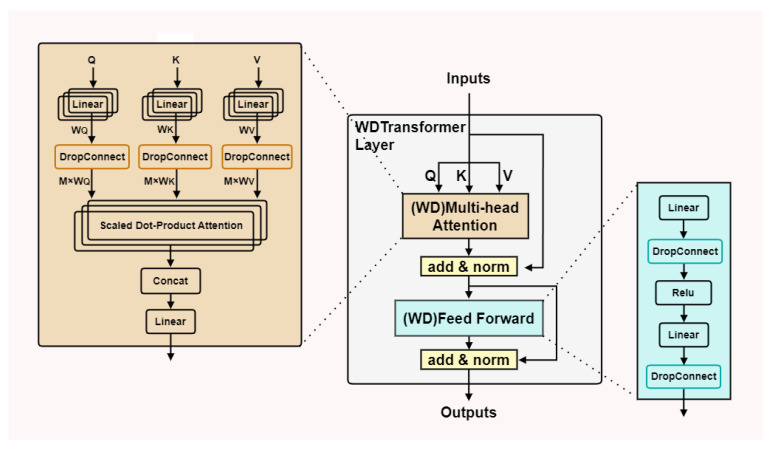
Weight-Dropped Transformer structure.

The Transformer model is a deep learning architecture that leverages self-attention mechanisms and is widely employed in sequence-to-sequence tasks [30]. The Transformer architecture comprises an encoder and a decoder, though only the encoder is utilized for the classification task in this study. The WDTransformer is an enhanced architecture derived from the Transformer encoder. The primary function of the encoder is to transform input sequences into context-aware deep feature representations. Each encoder layer consists of two key components: multi-head self-attention and a feed-forward neural network. The WDTransformer improves model regularization by incorporating DropConnect into both the multi-head attention mechanism and the feed-forward neural network. DropConnect is a generalized form of Dropout, where each weight connection is probabilistically dropped rather than dropping output units. In essence, DropConnect introduces dynamic sparsity at the weight level, whereas Dropout applies sparsity at the activation or output vector level. The structural details are illustrated in Figure 2.

In the traditional multi-head attention mechanism, the input Query (Q), Key (K), and Value (V) matrices are computed using weight matrices $W_{Q}$, $W_{K}$, and $W_{V}$, respectively. In the WDTransformer, DropConnect is incorporated after the application of weight matrices. This technique applies a random binary mask to the weight matrices, probabilistically dropping certain weights during training. This introduces dynamic sparsity at the weight level, effectively mitigating the risk of overfitting. The detailed computation process is outlined as follows:

(5) $$Attention(Q,K,V)=soft\max(\frac{(M\times W_{Q}){\left(M\times W_{K}\right)}^{T}}{\sqrt{d_{k}}})(M\times W_{V})V$$

where the encoding matrix M is responsible for dynamically modifying the weights transmitted during each training session, allowing the model to capture different combinations of features.

In the feed-forward neural network of the WDTransformer, DropConnect is also applied to the weight matrices of the fully connected layers, thereby enhancing the model’s regularization effect. The feed-forward network comprises two Dense layers with weight matrices $W_{1}$ and $W_{2}$. After applying the DropConnect mechanism, the feed-forward network computation can be expressed as follows:

(6) FFN(x)=ReLU((M1×W1)+b1)×(M2×W2)+b2

where encoding matrices $M_{1}$ and $M_{2}$ randomly drop weights, dynamically altering the connection patterns during each propagation. This enables the network’s output to rely on diverse stochastic connection combinations.

### 3.4. Combination Loss Function

In most intrusion detection applications, datasets commonly exhibit a long-tailed distribution, where minority class samples are significantly underrepresented compared to the majority. To simultaneously optimize classification accuracy and address the long-tail problem, this study integrates cross-entropy loss and focal loss. These two loss functions are combined using a weighted average, enabling the model to focus more effectively on minority class samples. The mathematical expressions for the combined loss, cross-entropy loss, and focal loss are presented below:

(7) $$L_{combined}=\beta \cdot L_{CE}+(1-\beta )\cdot L_{FL}$$

(8) $$L_{CE}=-\sum_{i=1}^{N}y_{i}\log(p_{i})$$

(9) $$L_{FL}=-\sum_{i=1}^{N}\alpha_{i}(1-p_{i}{)}^{\gamma }y_{i}\log(p_{i})$$

where, in Formula (7), $L_{CE}$ is the cross-entropy loss, $L_{FL}$ is the focal loss, and β is a weight coefficient used to control the ratio between cross-entropy loss and focal loss in the final combined loss. In Formula (8), N represents the total number of classes and $y_{i}$ represents the ground-truth label. $p_{i}$ denotes the model’s predicted probability for class i. In Formula (9), $\alpha_{i}$ is the weight factor for balancing different classes and γ is the tuning factor.

## 4. Proposed Model

**Figure 3:**
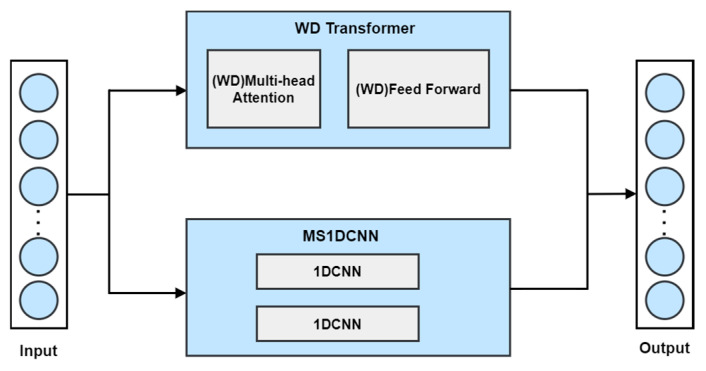
Algorithm overview flowchart.

This paper presents an approach to enhance intrusion detection in ICSs by leveraging a dual-channel feature extraction model, as illustrated in Figure 3. The proposed model is designed to improve the efficiency and accuracy of network intrusion detection in industrial control systems. It comprises two primary modules: MS1DCNN and WDTransformer. These modules are responsible for extracting local temporal features and global dependencies, respectively. The extracted features are subsequently combined in the feature fusion layer for classification.

Initially, the input data are processed through two parallel channels. The MS1DCNN module utilizes a multi-scale 1D convolutional network with kernel sizes 8 × 3 and 16 × 5. This module extracts fine-grained local temporal features from packet-level data. Following convolution, the features undergo dimensionality reduction using GlobalMaxPooling1D, which preserves critical information while reducing computational complexity.

Concurrently, the input data are processed by the WDTransformer module. This module, based on the Weight-Dropped Transformer, employs multi-head attention to capture long-range dependencies in the data. It effectively models global interactions. Additionally, the feed-forward network within this module enhances feature representation via nonlinear transformations.

After extracting both local and global features, the outputs of the two modules are integrated in the feature fusion layer. This integration produces a comprehensive feature vector. Finally, the comprehensive feature vector is passed through a fully connected layer for the classification of multiple network attack types.

The proposed model integrates the advantages of both local and global feature extraction. It captures diverse patterns in complex network attack scenarios, thereby enhancing detection accuracy and robustness.

**Figure 4:**
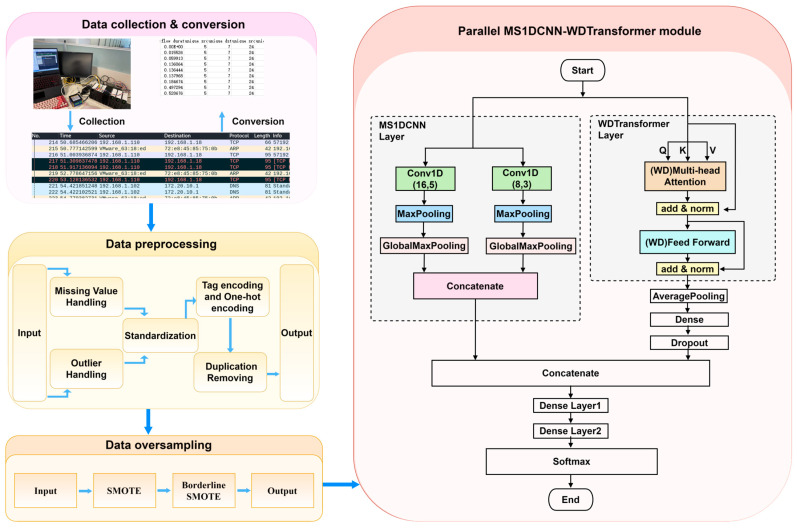
Detailed algorithm flowchart.

The proposed workflow for network intrusion detection comprises multiple steps, as illustrated in Figure 4. Initially, data are collected using network traffic capture tools, followed by protocol analysis and feature extraction. During preprocessing, tasks such as standardization, the handling of missing values, label encoding, and deduplication are carried out. To address class imbalance, SMOTE and Borderline-SMOTE are applied for oversampling, resulting in a balanced dataset. The model leverages parallel MS1DCNN and WDTransformer structures to extract local temporal features and global dependencies, respectively. Finally, feature fusion and a fully connected layer are utilized for classification, producing predictions across multiple attack classes. The subsequent sections provide a detailed explanation of each module and its specific role in the workflow.

### 4.1. Data Collection

**Figure 5:**
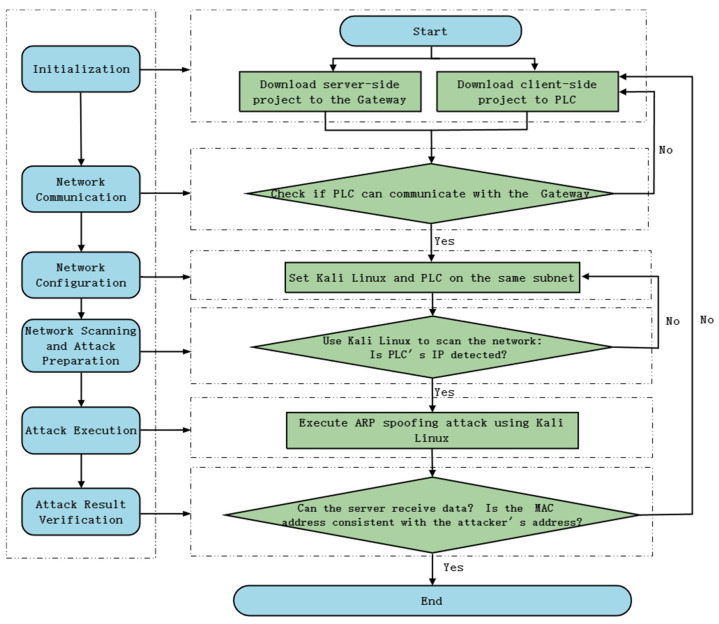
ARP attack flowchart.

To effectively evaluate the performance of the proposed network intrusion detection model, a dataset tailored for PLC network security in industrial control systems was constructed. The experiments were conducted using configuration software (Guoneng Xinkong, Beijing, China, Model: XinKong Xingtu First Edition) to set up the PLC (Guoneng Xinkong, Beijing, China, Model: P1000 module) and gateway (Guoneng Xinkong, Beijing, China, Model: D1800).Multiple typical network attack scenarios were designed and implemented in this study, including SYN-Flood, DDoS, Nmap, ARP, and Scapy attacks. These attacks encompass various network threats and address the primary security risks that industrial control systems may encounter. Taking the ARP attack as an example, the detailed experimental process is illustrated in Figure 5.

During data collection, various attacks were launched on the PLC module using the Kali Linux system. Simultaneously, Wireshark was employed to capture network traffic during the attacks. Each attack was conducted in an isolated environment, disconnected from the internet, to prevent interference or external threats to the PLC network.

The collected PCAP traffic data comprise a total of 369,940 entries. This dataset includes 159,600 normal traffic records, encompassing all modes of normal operation, and 210,340 attack traffic records, covering SYN-Flood, DDoS, Nmap, ARP, and Scapy attacks.

The raw dataset captured by Wireshark was stored in PCAP format, which could not be directly utilized. Consequently, the data from the PCAP files required extraction. The PCAP files were processed in Wireshark, where relevant feature fields were selected and exported, with the parsed results saved in CSV format.

**Figure 6:**
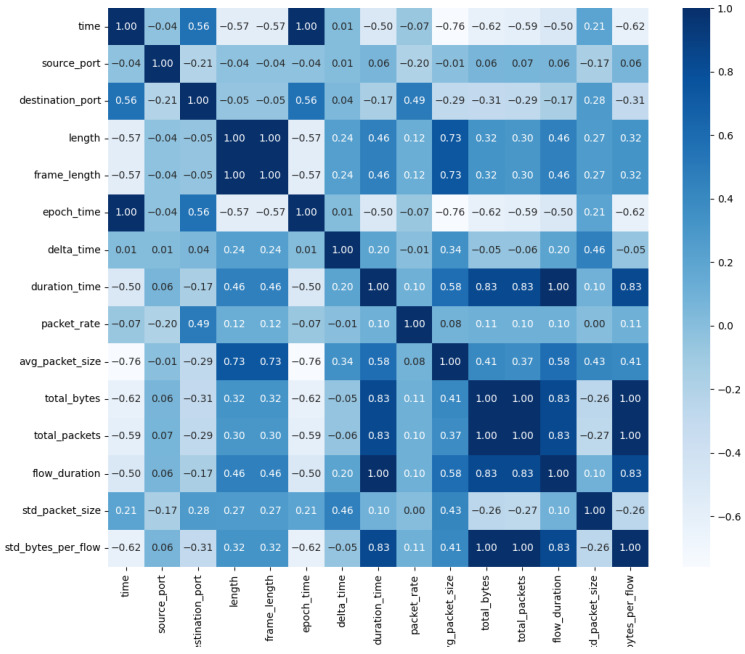
The heatmap of the correlation coefficients of input features.

Following feature selection, 15 significant feature fields were identified as the feature dimensions. These features were categorized into four groups: time-based features, network traffic features, packet size and traffic statistics, and derived features. Collectively, these features describe various attributes of network communication, offering valuable insights for intrusion detection. To further analyze the relationships between these features, a feature correlation heatmap was generated for visualization, as illustrated in Figure 6.

In the heatmap, color intensity reflects the strength of correlations between features. Darker colors indicate stronger correlations. Highly positively correlated features imply the presence of similar information, whereas negatively correlated features indicate an inverse relationship.

### 4.2. Data Pre-Processing

To ensure the model’s accuracy and robustness, the preprocessing of the CSV data was divided into five key steps: handling missing values, addressing outliers, data normalization, label encoding and One-Hot encoding and deduplication. Subsequently, the preprocessed dataset was split into training and testing sets, followed by oversampling.

1. Handling Missing Values: Features with a high proportion of missing values were removed to minimize their negative impact on the model. For features with fewer missing values, numerical values were imputed with the mean, and categorical values were imputed with the mode;
2. Handling Outliers: The parsed data were carefully inspected for errors, and entries with format inconsistencies, logical errors, or content anomalies were removed or corrected;
3. Data Normalization: To balance each feature’s contribution to model predictions and enhance overall accuracy, all data were normalized using the *z*-score method, as presented in the formula below:
  (10) $$z=\frac{x-\bar{x}}{\partial }$$
  where x represents the original data, $\bar{x}$ represents the mean value of the corresponding feature column in the original data, and ∂ represents the standard deviation of the original data;
4. Label Encoding and One-Hot Encoding: For categorical features, such as target labels, label encoding was initially applied to transform categorical labels into numerical format. Subsequently, One-Hot encoding was applied to these numerical labels, transforming each label into a binary vector, where a single element is set to 1 and the remaining elements are 0;
5. Data Deduplication: Duplicate samples in the dataset were identified and removed to mitigate bias and reduce the risk of overfitting caused by redundant data.

Following data processing, 349,346 traffic samples were retained in the final dataset. This dataset includes 150,000 samples of normal behavior, encompassing all modes. It also contains 199,346 attack samples, comprising 102,530 SYN-Flood attacks, 90,727 DDoS attacks, 2442 Nmap attacks, 2450 ARP attacks, and 1242 Scapy attacks.

### 4.3. Data Oversampling

The dataset used in this study exhibits a significant imbalance. The number of Nmap, ARP, and Scapy attack samples is considerably smaller compared to other attack types and normal traffic. This imbalance can lead traditional machine learning models to favor majority classes, thereby neglecting the features of minority classes. Consequently, SMOTE and Borderline-SMOTE were applied to oversample the minority attack samples.

Prior to oversampling, the dataset was divided into training and testing sets, with 80% allocated to training and 20% to testing. The training set was utilized to train and fine-tune the model, while the testing set was employed to evaluate its final performance. Stratified sampling was employed to ensure adequate representation of minority classes in both sets. Table 1 presents the distribution of the training and testing sets.

The minority class samples in the training set were oversampled. Initially, new minority class samples were generated in the feature space using SMOTE. Subsequently, Borderline-SMOTE was applied to further oversample boundary samples, improving the model’s capability to recognize them. Table 2 presents the distribution of the training set before and after oversampling.

After oversampling, the number of minority class samples in the training set increased substantially. To mitigate the overfitting caused by excessive synthetic samples and to preserve data diversity and authenticity, the number of minority samples was kept relatively low. This strategy addresses class imbalance while maintaining model robustness and training efficiency.

### 4.4. MS1DCNN WDTransformer Module

The parallel MS1DCNN–WDTransformer module comprises two branches: the MS1DCNN branch and the Weight-Dropped Transformer branch. The MS1DCNN module utilized in this study adopts a parallel branch structure with two different convolution kernel sizes. The first layer comprises 8 kernels of size 3, while the second layer consists of 16 kernels of size 5. After each convolution, a ReLU activation function is applied to perform non-linear mapping. The corresponding calculation formula is provided below:

(11) ReLU(x)=max(0,x)

Each convolution branch is followed by MaxPooling and GlobalMaxPooling operations. MaxPooling is utilized for downsampling to extract critical local features, while GlobalMaxPooling reduces the time steps while retaining essential global features. Following pooling, the outputs of the convolution branches are concatenated to integrate features from different kernels, forming the final feature representation. The convolution stride is set to 1, and the pooling window size is configured to 2.

The WDTransformer module initially extracts global features from the input sequence using WD-Multi-Head Attention, enhancing the model’s capability to capture long-range dependencies. Subsequently, WD-Feed Forward applies non-linear transformations to these features, enhancing the model’s generalization and robustness through DropConnect. AveragePooling is utilized to further compress feature dimensions. The compressed features are subsequently mapped by a Dense layer, followed by a Dropout layer to mitigate the risk of overfitting. In WD-Multi-Head Attention, each attention head is configured with a size of 16. DropConnect is assigned a regularization rate of 0.6, and ReLU serves as the activation function for non-linear mapping.

Finally, the outputs of the MS1DCNN and WDTransformer branches are fused through a Concatenate layer. The fused features are subsequently passed through two fully connected layers, comprising 64 and 32 neurons, respectively, both employing ReLU as the activation function. The final output is generated through a Softmax activation function, yielding the predicted probabilities for multiple classes. The corresponding calculation formula is provided below:

(12) $$Soft\max(z_{i})=\frac{e^{z_{i}}}{\sum_{j=1}^{K}e^{z_{i}}}$$

where $z_{i}$ is the input value and K is the number of classes. The Softmax function normalizes the predicted class probabilities to the range [0, 1], covering multiple classification tasks, including Normal, SYN-Flood, DDoS, Nmap, ARP, and Scapy.

This section employs a custom combined loss function that integrates cross-entropy loss and focal loss. The focal loss function incorporates two parameters, alpha and gamma, to adjust the weighting of positive and negative samples and to emphasize hard-to-classify samples. The parameter alpha is set to 0.25 to enhance the model’s focus on minority samples, while gamma is set to 2.0 to amplify the penalty for hard-to-classify samples. Additionally, the parameter beta regulates the balance between cross-entropy loss and focal loss, with a value of 0.5 employed in this study to achieve a balanced integration.

### 4.5. Performance Metrics

In this section, multiple commonly used classification evaluation metrics are used to assess the model’s performance, including accuracy, precision, recall, F1-Score, FNR, and FPR. These metrics provide a comprehensive evaluation of the model’s performance in classification tasks.

- Accuracy represents the proportion of correctly predicted samples to the total number of samples:

(13) $$Accuracy=\frac{TP+TN}{TP+TN+FP+FN}$$

- Precision represents the proportion of correctly predicted positive samples out of all predicted positives:

(14) $$Precision=\frac{TP}{TP+FP}$$

- Recall represents the proportion of actual positive samples that are correctly predicted:

(15) $$Recall=\frac{TP}{TP+FN}$$

- F1-Score represents the harmonic mean of recall and precision, representing a balance between the two:

(16) $$F1=\frac{2*Precision*Recall}{Precision+Recall}$$

- False Negative Rate (FNR) represents the proportion of actual positive samples incorrectly predicted as negative:

(17) $$FNR=\frac{FN}{FN+TP}$$

- False Positive Rate (FPR) represents the proportion of actual negative samples incorrectly predicted as positive:

(18) $$FPR=\frac{FP}{FP+TN}$$

TP represents the number of true positives, FN represents false negatives, FP represents false positives, and TN represents true negatives.

## 5. Experiments and Results

### 5.1. Experimental Environment

The experiments in this study were conducted using Python 3.9.18, with TensorFlow serving as the deep learning framework. The operating system was Windows 10, equipped with an Intel Xeon Silver 4210R processor, 128 GB of RAM, and an NVIDIA RTX A5000 GPU featuring 24 GB of VRAM.

### 5.2. Experimental Results and Analysis

**Figure 7:**
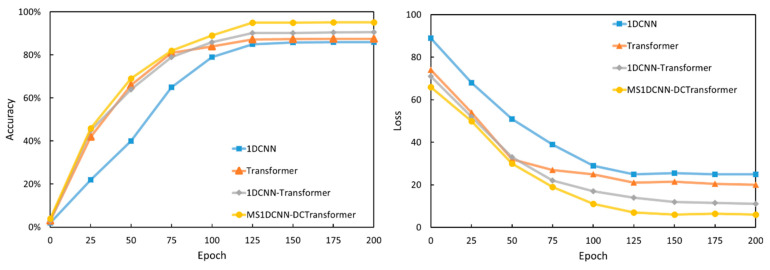
Accuracy and loss curves.

After data preprocessing and oversampling, the dataset was prepared for training the deep learning models. Multiple experiments were conducted on four models—1DCNN, Transformer, 1DCNN-Transformer, and MS1DCNN-WDTransformer—using identical model parameters. The average results of 10 experimental runs were computed, with the training phase spanning 200 epochs. Figure 7 illustrates the trends in model accuracy and loss across epochs.

During the initial 10 to 30 epochs, all models exhibited rapid improvement. Accuracy improved significantly, while loss declined rapidly. The 1DCNN model, in particular, exhibited considerable fluctuations during the early fitting process, with accuracy ranging from 20% to 50% and loss fluctuating between 50 and 80. In contrast, the MS1DCNN-WDTransformer model demonstrated a faster convergence rate, achieving over 70% accuracy and reducing the loss to below 30 by the 30th epoch. Between approximately 50 and 100 epochs, all models began to converge, achieving accuracies exceeding 80%. The MS1DCNN–WDTransformer outperformed other models, reaching over 90% accuracy and reducing the loss to below 20 by the 100th epoch. Around 150 epochs, the loss for training, validation, and test sets continued to decline, while accuracy steadily improved, indicating stable performance. To ensure better convergence, the training process was extended to 200 epochs. By the end of 200 epochs, the MS1DCNN–WDTransformer model achieved an accuracy of nearly 95%, with a stable loss of approximately 6, significantly outperforming other models. The model’s advantages on other metrics are summarized in Table 3, where lower FNR and FPR values indicate superior classification performance.

**Figure 8:**
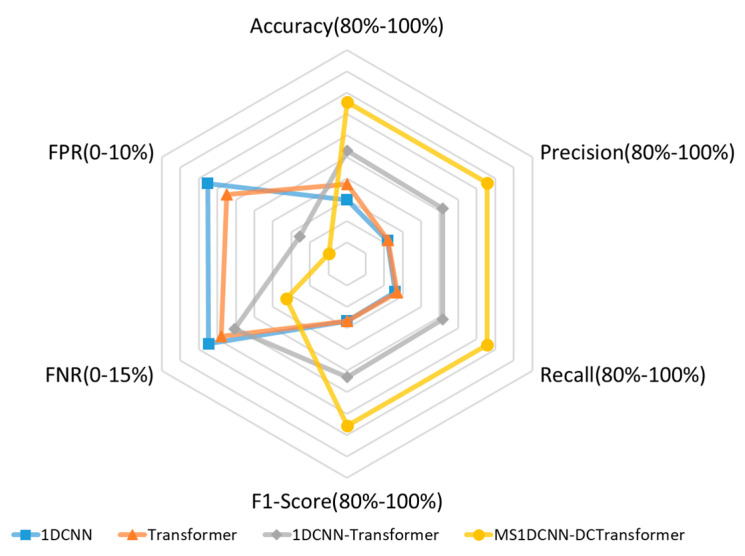
Experimental results radar chart.

Table 3 reveals that the 1DCNN model, which relies solely on local convolutional feature extraction, exhibits relatively low classification performance, achieving an accuracy of 0.8598 and an F1-Score of 0.8534. In contrast, the Transformer model, incorporating the self-attention mechanism, achieves superior global feature extraction, yielding improved performance with an accuracy of 0.8746 and an F1-Score of 0.8533. The parallel 1DCNN-Transformer model combines both techniques, further enhancing classification performance to achieve an accuracy of 0.9056 and an F1-Score of 0.9058. This underscores the effectiveness of integrating convolutional features with the self-attention mechanism to enhance classification accuracy. The proposed MS1DCNN–WDTransformer model surpasses all other models across all evaluation metrics. Specifically, it achieves an accuracy of 0.9511 and an F1-Score of 0.9512, markedly outperforming the other models. In handling complex network attacks, the MS1DCNN–WDTransformer demonstrates remarkable robustness, achieving an FNR of 0.0489 and an FPR of 0.0098, values considerably lower than those of other models. These results confirm that the MS1DCNN–WDTransformer exhibits significant advantages in classification performance, generalization ability, and robustness in handling complex network attacks, establishing it as a more effective and reliable classification model. To provide a more intuitive comparison, the data are visualized using a radar chart, as illustrated in Figure 8.

Table 4 summarizes the experimental results of the proposed MS1DCNN–WDTransformer model in comparison with two traditional classification models, SVM and XGBoost, and three deep learning models, Attention-based CNN–LSTM, CNN–GRU, and DBN, on an imbalanced dataset.

Traditional methods demonstrated poor performance on imbalanced datasets. The F1-Scores of SVM and XGBoost were 0.8533 and 0.8754, respectively. Both models exhibited high misclassification rates. XGBoost recorded a false negative rate of 0.0956, which limited its practical applicability.

Deep learning models achieved superior performance. The Attention-based CNN–LSTM model yielded an F1-Score of 0.9130 and a recall of 0.9128, employing convolution to extract spatial features and LSTM to capture temporal dependencies. However, it still exhibited room for improvement, with a false negative rate of 0.0683 and a false positive rate of 0.0300. CNN–GRU and DBN demonstrated further improvements in classification results. DBN yielded an F1-Score of 0.9328, demonstrating a stronger capacity to capture complex features.

The proposed MS1DCNN–WDTransformer model surpassed all other models across every metric. It achieved an F1-Score of 0.9512, with precision and recall both reaching 0.9511. The model exhibited significantly lower misclassification rates, achieving a false negative rate of 0.0489 and a false positive rate of 0.0098. This superior performance can be attributed to several factors: hybrid oversampling improved data balance, multi-scale convolutional layers enhanced feature extraction, DropConnect regularization strengthened generalization, and the WDTransformer’s self-attention mechanism effectively captured long-range dependencies. These elements collectively enabled the model to excel in complex and imbalanced multi-class classification tasks.

Table 5 summarizes the experimental results of the proposed model compared to several baseline models on the publicly available WADI dataset, highlighting its effectiveness and generalizability. The WADI dataset, derived from a real-world water distribution system, simulates components like water tanks, valves, and pumps, alongside sensors’ monitoring flow rate, water levels, and pressure. It includes approximately 1.2 million time-series records (sampled at 1-s intervals) across 123 features, making it highly complex with significant temporal dependencies. However, its pronounced imbalance, with normal data vastly outnumbering attack data, poses challenges for effective anomaly detection.

The results show that the proposed MS1DCNN–WDTransformer model outperforms all baseline models across all metrics. It achieves exceptionally low FNR and FPR values of 0.0468 and 0.0012, indicating minimal missed detections and false alarms. These results demonstrate the model’s robust detection capability and adaptability to complex industrial environments, effectively handling various attack scenarios in the WADI dataset. This highlights its strong potential for intrusion detection in industrial control systems.

**Figure 9:**
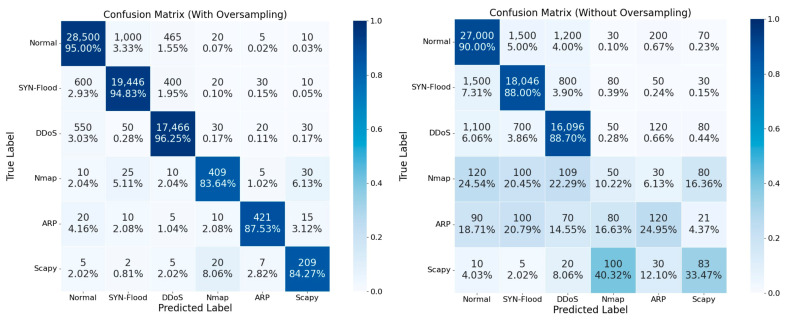
Confusion matrices with and without the use of oversampling.

To validate the effectiveness of the SMOTE–Borderline-SMOTE method in mitigating class imbalance, the model’s classification performance was compared before and after oversampling. Figure 9 presents confusion matrices before and after applying oversampling. The results clearly indicate that, after applying the oversampling technique, the classification performance of minority classes improved significantly. Specifically, the correct recognition rate of the Scapy class increased from 33.47% to 84.27% following oversampling. The performance of the Nmap and ARP classes also improved, with recall rates rising from 40.32% to 83.64% and 87.53%, respectively. The SYN-Flood and DDoS classes exhibited notable improvements as well, with accuracies rising from 88.00% and 88.70% to 94.83% and 96.25%, respectively. The SMOTE–Borderline-SMOTE technique not only enhanced the classification performance of minority classes but also substantially reduced inter-class misclassification. Overall classification accuracy improved substantially, with notable gains for minority classes.

## 6. Conclusions

This study introduces a novel intrusion detection model for ICSs, integrating MS1DCNN and WDTransformer to effectively address key challenges in PLC network security. The WDTransformer module incorporates a novel regularization method, DropConnect, into both the attention and feed-forward components of the Transformer architecture. This innovation mitigates the risk of overfitting while enhancing the model’s capacity to capture global dependencies in network traffic, enabling more robust and accurate intrusion detection. Meanwhile, the dual-channel architecture of the proposed model integrates MS1DCNN and WDTransformer to extract complementary features. MS1DCNN captures fine-grained local temporal features, while WDTransformer models long-range dependencies. This integrated design ensures a comprehensive representation of both local and global patterns in the data, significantly enhancing classification accuracy.

To address the pervasive issue of class imbalance in ICS datasets, this study employs a hybrid oversampling approach that combines the SMOTE and Borderline-SMOTE techniques. This method not only improves the representation of minority classes but also substantially reduces misclassification rates. The hybrid oversampling strategy was validated on both the self-constructed ICS dataset and publicly available imbalanced datasets, demonstrating its effectiveness in enhancing model performance across diverse datasets. Experimental results indicate that the proposed model achieves an accuracy of 95.11% and an F1-Score of 95.12%, surpassing traditional machine learning and deep learning models. It demonstrates superior performance in detecting various types of network attacks, particularly those in minority categories.

The methodologies and findings in this study have practical implications for improving intrusion detection systems in industrial environments. By addressing class imbalance and optimizing long-tail distributions, the proposed model is well-suited for deployment in real-world ICS scenarios, where data distributions are inherently imbalanced and diverse attack types must be reliably detected. Furthermore, the integration of multi-scale feature extraction and hybrid oversampling techniques provides a foundational framework for the development of next-generation intrusion detection systems. This framework serves as a foundation for further research, including the exploration of lightweight models, real-time detection systems, and techniques for detecting unknown attacks to address evolving industrial security needs.

In summary, this study contributes to the advancement of ICS network security by presenting a robust intrusion detection model that addresses key challenges such as data imbalance and long-tail distributions. Future work will focus on further optimizing the model’s structure to improve computational efficiency and adaptability. Lightweight models will be explored to meet the real-time requirements of industrial environments, while techniques such as self-supervised learning and domain adaptation will be investigated to enhance the model’s versatility and robustness in dynamic and complex industrial scenarios.

## Figures and Tables

**Table 1:** The different classes of attacks in the dataset.

Class Name	Training Set (80%)	Testing Set (20%)
Normal	120,000	30,000
SYN-Flood	82,024	20,506
DDos	72,581	18,146
Nmap	1953	489
ARP	1924	481
Scapy	994	248
Total	279,476	69,870

**Table 2:** Distribution of training set before and after oversampling.

Class Name	Before Oversampling (%)	After Oversampling (%)
Normal	42.93%	33.57%
SYN-Flood	29.35%	22.95%
DDos	25.97%	20.31%
Nmap	0.70%	9.29%
ARP	0.70%	9.15%
Scapy	0.36%	4.73%
Total	100%	100%

**Table 3:** The experimental results of each method.

Method	Accuracy	Precision	Recall	F1-Score	FNR	FPR
1DCNN	0.8598	0.8436	0.8519	0.8534	0.1120	0.0752
Transformer	0.8746	0.8441	0.8539	0.8533	0.1019	0.0651
1DCNN–Transformer	0.9056	0.9030	0.9033	0.9058	0.0910	0.0255
MS1DCNN–WDTransformer	0.9511	0.9514	0.9511	0.9512	0.0489	0.0098

**Table 4:** The experimental results of comparative experiments using the self-constructed dataset.

Method	Accuracy	Precision	Recall	F1-Score	FNR	FPR
SVM	0.8746	0.8441	0.8539	0.8533	0.0905	0.0610
XGBoost	0.8769	0.8745	0.8635	0.8754	0.0956	0.0589
Attention-based CNN–LSTM	0.9126	0.9188	0.9128	0.9130	0.0683	0.0300
CNN–GRU	0.9290	0.9318	0.9222	0.9317	0.0685	0.0297
DBN	0.9330	0.9320	0.9290	0.9328	0.0675	0.0292
MS1DCNN–WDTransformer	0.9511	0.9514	0.9511	0.9512	0.0489	0.0098

**Table 5:** The experimental results of comparative experiments using the WADI dataset.

Method	Accuracy	Precision	Recall	F1-Score	FNR	FPR
SVM	0.8559	0.8254	0.8003	0.8116	0.1997	0.0453
XGBoost	0.8806	0.8401	0.8208	0.8305	0.1792	0.0384
Attention-based CNN–LSTM	0.9253	0.9123	0.9004	0.9064	0.0996	0.0280
CNN–GRU	0.9301	0.9205	0.9152	0.9173	0.0848	0.0246
DBN	0.9002	0.8903	0.8850	0.8877	0.115	0.0302
MS1DCNN–WDTransformer	0.9587	0.9606	0.9572	0.9606	0.0468	0.0012

## Data Availability

Data are contained within the article.
